# Evaluating the Rates of Mortality and Cardiac Catheterization Using High-Sensitivity Troponin and Conventional Troponin Assays

**DOI:** 10.7759/cureus.63538

**Published:** 2024-06-30

**Authors:** Agostino Grittani, Saul Ramos, Chabelly Gomez, Cesar A Varela, Nikhil Puri, Samantha Zarry, Jose H Suarez

**Affiliations:** 1 Internal Medicine, St. George's University, Toronto, CAN; 2 Internal Medicine, University of Medicine and Health Sciences, Saint Kitts, KNA; 3 Research, Keralty Hospital, Miami, USA; 4 School of Medicine, St. George's University, St. George, GRD; 5 Health Sciences, The University of Western Ontario, Toronto, CAN; 6 Family Medicine, Keralty Hospital, Miami, USA

**Keywords:** retrospective studies, high sensitivity cardiac troponin (hs-ctn), nste-acs, cardiac troponin, non-st segment elevation myocardial infarction (nstemi)

## Abstract

Non-ST segment elevation myocardial infarction (NSTEMI) is an acute coronary syndrome event where myocardial ischemia is present, with an increase of cardiac troponins without an elevation of the ST segment. One of the fundamental measures used to diagnose or rule out acute coronary syndrome (ACS) is troponin levels in the blood. Troponin is a broad term used for the category of muscle contraction regulatory proteins and is commonly measured during ACS evaluation. Troponin I is only released by cardiac tissue, while some assay measurements will also pick up troponin released by skeletal muscle injury. This retrospective observational study was performed investigating troponin assays and how they relate to patient’s outcomes. The troponin assays used in this Miami hospital where the database of patients was collected between 2018 and 2023 were troponin I (cTnI), the conventional troponin assay, and the newer high-sensitivity troponin I assay (hs-cTn). In this observational study patients who received an admitting diagnosis of NSTEMI corroborated by an independent cardiologist had their respective troponin assay levels included. Patients found to have ECG changes significant for non-ischemic pathologies, or echocardiogram findings suggestive of myocardial dysfunction not clinically correlated to an ACS were excluded from the study. A total of 75 patients were included in this study and the mean age was 75.97 ±14.72 years, with a presentation of chest pain, dyspnea and general weakness recorded in 59% (n = 45) of patients. The median time between troponin samples was 6.63 hours across both assays and hs-cTn showed a 4.99% increase in variation between samples while cTnI had a decrease of 2.53%. The study objective is to support whether there is a difference in rates of cardiac catheterization or mortality based on the type of troponin testing. There was no significant association found between, the type of troponin assay used during hospital admission, and the outcomes of catheterization and death (p > 0.009).

## Introduction

Acute coronary syndrome (ACS) includes non-ST segment elevation Myocardial Infarction (NSTEMI), unstable angina (UA), and STEMI. In 2000, the European Society of Cardiology along with the American Heart (AHA) and the World Health Organization came to a consensus on the definition of infarction [1]. An acute myocardial injury with clinical evidence of ischemia illustrated as an elevated troponin measurement at or above the 99th percentile of the upper reference limit (URL), with elevations ≥1 mm (0.1 mV) in anatomically contiguous leads or ≥2 mm (0.2 mV) in leads V2 and V3 [1]. This is established by measuring the troponin values in a health control population and comparing it to the patient undergoing a possible ACS and determining whether their troponin level is higher than 99% of the healthy population [1].

The exponential rise in troponin concentrations in the blood during an infarction event is a commonly used indicator for diagnosis of a possible ACS. High-sensitivity cardiac troponins (hs-cTn) are organ-specific, so the presence of troponins at the clinically significant range can confidently diagnose a patient with an infarction variant. A specific troponin value denotes a definitive diagnosis of NSTEMI/STEMI, and factors such as age, gender, ethnicity, and comorbidities impact troponin values [2]. It is imperative to collect additional patient history and presenting symptoms to help differentiate an ACS from other acute/chronic illnesses, such as heart failure, sepsis, and chronic kidney disease, that can be associated with elevated troponins [2].

ACS is responsible for 780,000 hospital admissions every year, with an estimated 70% of these cases attributed to NSTEMI in the United States [3]. These acute cardiac events have a significant morbidity and mortality rate that requires a more comprehensive diagnostic picture to treat patients; accordingly, while lowering the risk of future infarctions [3,4]. This retrospective observational study explores the correlation between cardiac biomarkers and patient outcomes. The objective is to investigate whether there is a measurable difference between patients needing cardiac catheterization or expiring when comparing the different types of troponin assays used. Secondary objectives include expanding the existing literature on NSTEMI patients and a more succinct clinical profile of NSTEMI. Further research is warranted to validate and expand upon the results found in this study.

## Materials and methods

75 patients with a diagnosis of NSTEMI in the ER at Keralty Hospital located in Miami, USA were collected from June 2018 to September 2023. Included in this retrospective observational study were patients 18 years of age or older, who had elevated troponin levels in their blood measured via hs-cTn assay testing or conventional cTn testing. Troponin baseline values were obtained within the first hour of patient admission, and these values along with the absence of non-ST segment elevation were recorded. All patients who had elevated troponin values on admission were seen by an independent cardiologist who determined the presence of an ACS event before inclusion in the study. Patients determined to have non-ischemic changes in the myocardium unrelated to coronary perfusion on echocardiogram or ECG testing were excluded from this study. Cardiac pathologies that can increase troponin levels and be identified on cardiac testing like a left bundle branch, decompensated heart failure, pulmonary embolism or myocarditis were excluded from this study. The data collected on the 75 patients in this study included vital signs, initial patient presentation symptoms, comorbidities, troponin I (cTnI) and high-sensitivity troponin I (hs-cTn) values, time elapsed between troponin measurements, echocardiogram findings, ECG findings and patient discharge outcomes. Patients were separated into two groups dependent on the troponin test they received. The troponin assay used denoted cTn/ TnI had a reference normal interval of 0.000 to 0.056 ng/mL and a critical value of 0.599 ng/ml. The reference normal interval of hs-cTn is 0 to 51.4 ng/L, with a critical value of 51.5 ng/L. Significance was assessed with a threshold of p < 0.05 and STATA 17 (StataCorp, College Station, USA) and GraphPad Prism Version 10.00 (GraphPad, San Diego, USA), for analysis.

## Results

**Figure 1:**
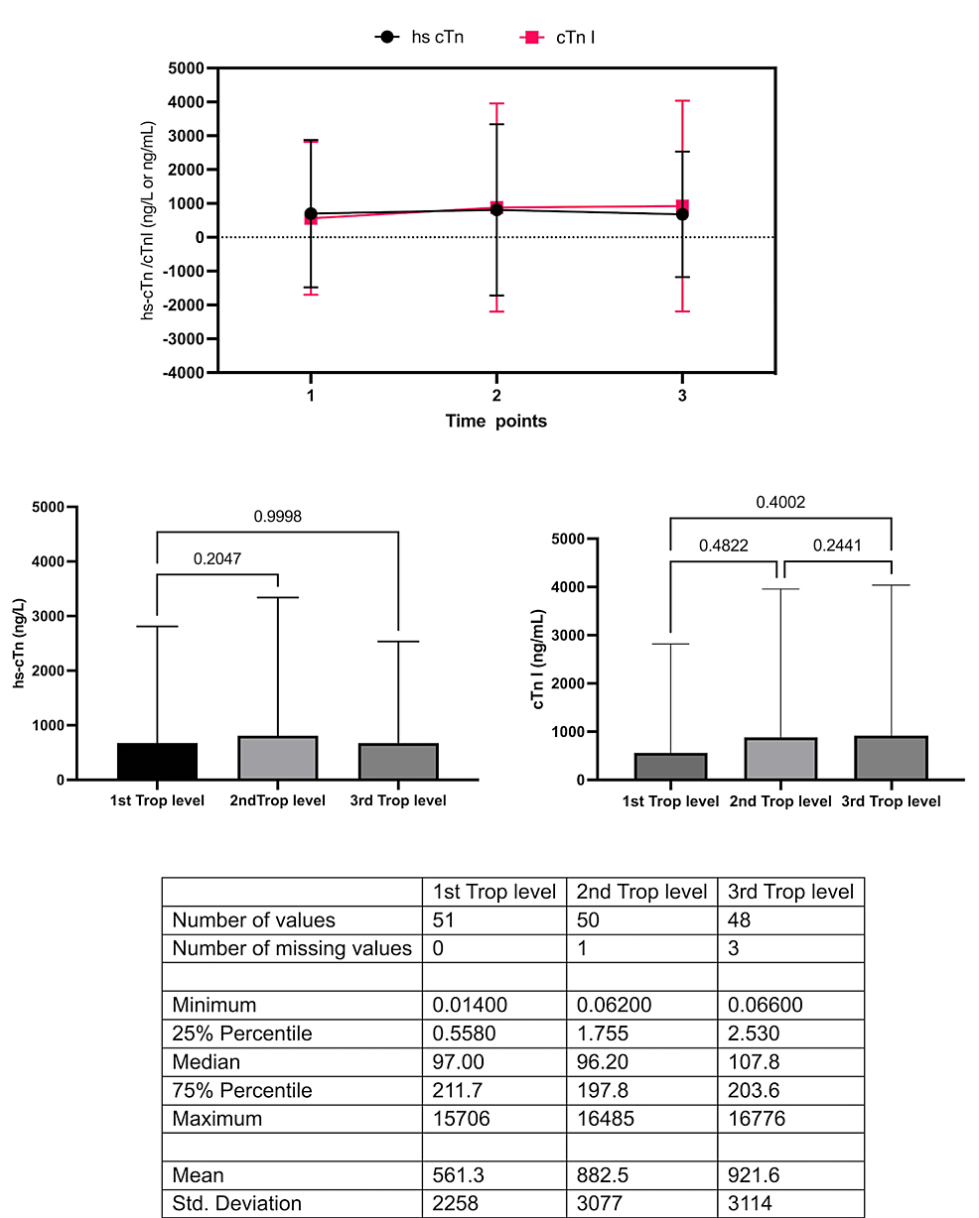
Variation of troponin levels over time points hs cTnI: high-sensitivity troponin I; cTnI: troponin I

Regarding the population distribution, 51% (n = 38) of patients were female, the mean age was 75.97 ± 14.72 years, and the average body mass index was 27.55. In terms of relevant medical history findings, 10.67% (n = 8) patients had experienced stroke, 5.33% (n = 4) pulmonary embolism, and 8.86% (n= 6) cardiomegaly The most common collection of symptoms seen at initial presentation included chest pain, dyspnea, and general weakness, identified in 59% (n=45) of patients. Median heart rate was 82, oxygen saturation 97%, hemoglobin 11.9 g/dl, and estimated glomerular rate (eGFR) 62 ml/min. The most common echocardiogram finding was ventricular hypertrophy with preserved left ventricular systolic function seen in 44.4% (n=33). In this study, hs-cTn was used in 68% (n=51) and cTnI in 32% (n=24), the median time between the first and second troponin samples in both groups was 6.63 hours. The percentage of variation between time points showed a decrease of 2.53% cTnI and an increase of 4.99% hs-cTn as seen in Figure 1. Approximately 16.7% (n = 4) of the patients who underwent cTnI testing received catheterization, while 33.33% (n = 17) of those who underwent hs-cTn received catheterization. Moreover, 4.17% (n = 1) of those who underwent cTnI testing died, and 13.73% (n = 7) of those who underwent hs-cTn testing died. There was no significant association between, the type of troponin assay and the outcomes of catheterization and death (p > 0.009).

## Discussion

The diagnostic criteria subclasses of ACS are clear, but the variability in patient presentations and ambiguous lab findings, make accurate diagnoses of cardiac conditions difficult. The innovation of assays that measure cardiac muscle tissue proteins unique to the heart helps physicians distinguish between patients who have similar initial presentations. Although these assays are a great addition to the diagnosis of ACS conditions, doctors ultimately use ECG findings to dictate management with angiography, often before troponins have reached significant levels in the blood [5,6]. Patients exhibiting atypical symptoms and signs are also increasingly prevalent in the elderly illustrating the importance of ECG presentation as the most important tool for a diagnosing physician [5,6]. ECGs yield important information, but they are not without their shortcomings. Diagnosis of NSTEMI/STEMI relies on the deviation of the ST segment level away from the preceding P-R segment and/or the following T-P segment, denoted as being non-isoelectric [5,6]. This definition fails to acknowledge deviations of the ST segment that are non-ischemic in origin, which on average can be found in 15% of the general population. 91% of the men who serve in the United States between the ages of 18 and 58, without any registered, or prior cases of diagnosed heart conditions were found to have ST segment variations [7]. This is one of the many reasons STEMI guidelines have varied ECG criteria depending on gender due to differences in ST segment deviations [8,9]. The decrease in ST segment elevation as patients age, and the documented differences in the magnitude of ST elevations seen in different ethnic backgrounds, impact the reliability of using ECGs when diagnosing infarction [8-10].

These findings support a clinical environment where troponin assays play a more significant role in the diagnostic work-up of ACS. The objective of this study was to showcase the potential difference in utility and effectiveness between hs-cTn and cTnI in ACS while evaluating the limitations of troponin assays. This investigation collected data surrounding the timing interval between troponin measurements, the patient's discharge outcomes, and catheterization or mortality rates based on the type of troponin assay used. Although the results of the study suggested mortality or cardiac catheterization rates were not impacted by the type of troponin assay used; other studies have highlighted benefits unique to high-sensitivity troponin assays.

**Figure 2:**
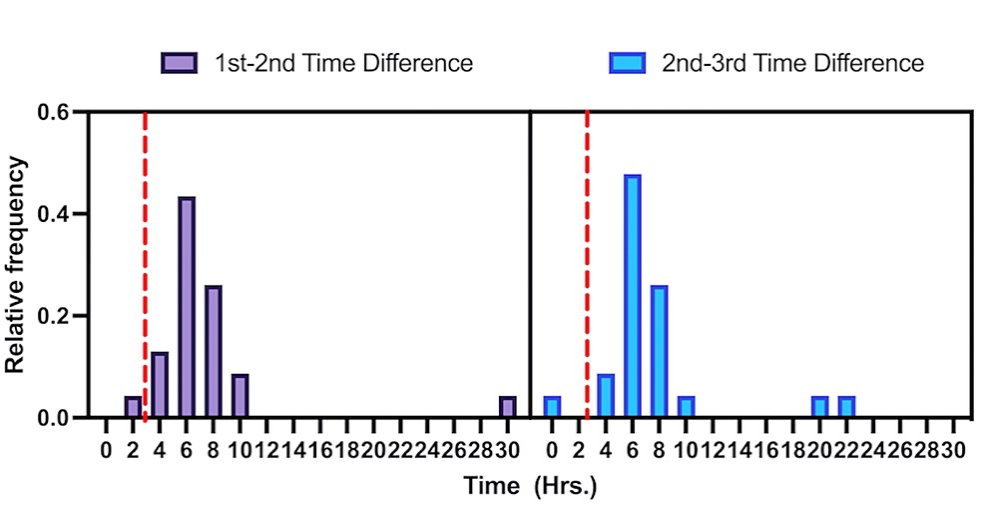
Time frequency distribution between cardiac measurement time points and healthy population reference for acute coronary syndrome.

The hs-cTn assay detects smaller concentrations of troponin in the blood, and Reichlin et al. demonstrated increases in sensitivity and negative predictive value (NPV) in regard to patients admitted for ACS [11]. This increase in sensitivity is appreciated when comparing the difference in troponin assay cut-off points illustrated in Figure 2 [9]. A prospective multicenter observational study collected information from patients admitted with chest pain as their chief complaint and worry of potential ACS. Hs-cTn testing initially and after one hour was used to investigate the potential utility of hs-cTn [11]. This type of troponin assay had a 100% sensitivity rate among participants. These disease screenings were further confirmed when ECG was evaluated and carbureted by independent cardiologists who were blinded to the troponin results [11]. Accurately determining infarction events in patients with potential ACS, illustrates the utility of high-sensitivity troponin as a tool. The same study reported an 84% positive predictive value (PPV) for elevated troponins indicating a positive ACS event [11].

The concept is that the utility of troponin testing, whether at initial presentation, or one hour after admission, is the ability to infer that without elevated troponin levels in the blood, the likelihood patient presenting with acute chest pain, having an ACS occurrence is extremely low. Another takeaway from the study is that elevated troponin levels in the blood equate to a diagnosis of STEMI/NSTEMI, because with an 84% PPV, 16% of patients had non-cardiac causes of myocardial ischemia such as a history of renal failure, hypovolemia, sepsis, acute strenuous exercise, or severe motor vehicle accident [11,12]. 

**Figure 3:**
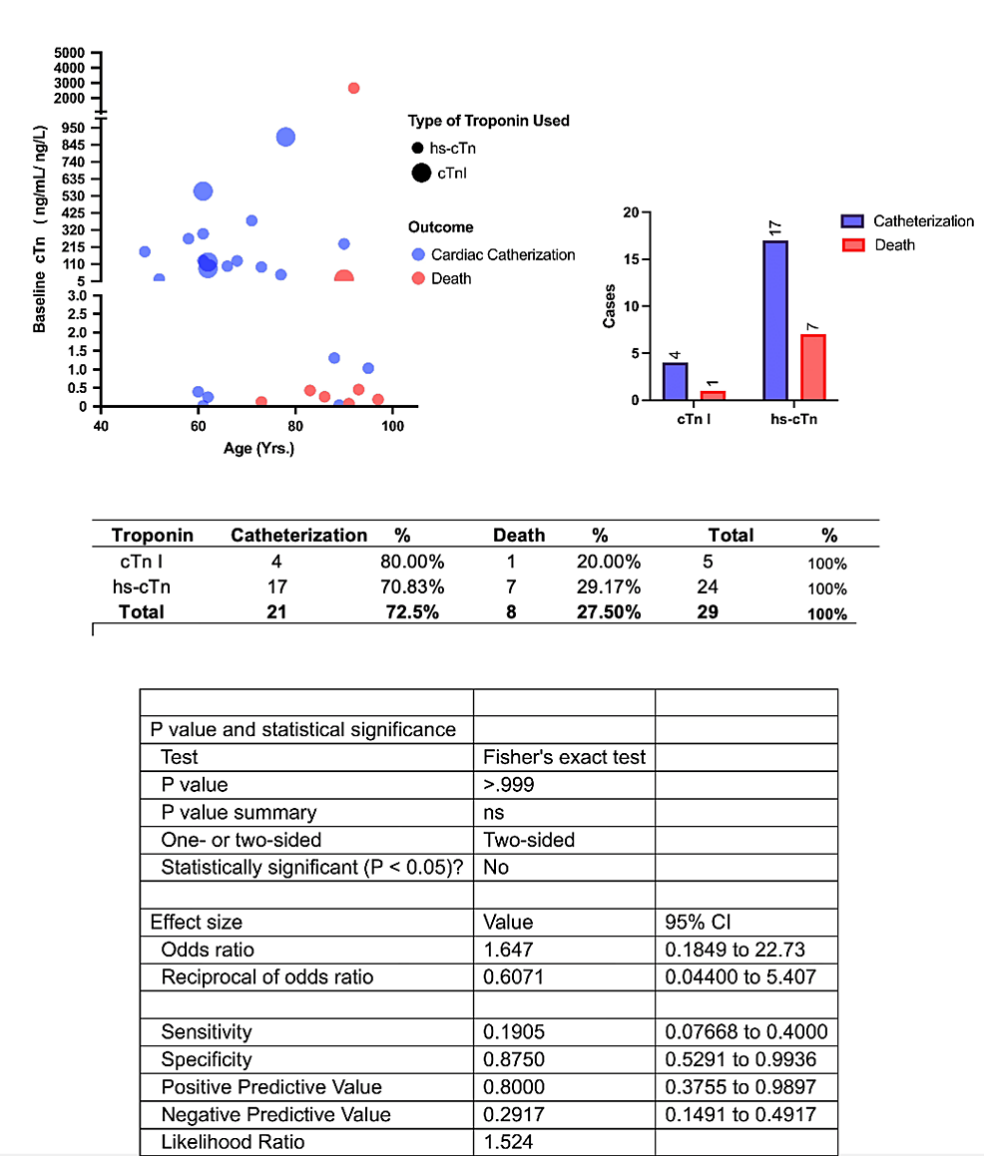
Relationship between cardiac troponin measurement and outcomes. hs-cTnI: high-sensitivity troponin I, cTnI: troponin I

The Gutenberg health study retroactively examined troponin levels in the general population and measured 4,138 subjects with the troponin 1 immunoassay (Abbott ARCHITECT STAT version; Abbott, Chicago, USA) [13]. The 99th percentile of the overall population was 27 ng/L, but this metric failed to account for multiple comorbidities in a given patient. Changes in the structure and function of the heart influence the criteria for diagnosing ACS conditions on ECG, but there is a lack of knowledge about normalized troponin parameters and what deviations should be considered significant enough to diagnose NSTEMI or STEMI. When the Gutenberg Study examined the patient population and excluded pre-existing cardiac abnormalities; or elevated natriuretic peptide levels, the 99th percentile values were considered to be statistically significant with much lower levels of troponin [13]. The study’s conclusion supported the idea that age, gender, and structural/functional cardiac abnormalities have a significant influence on the levels measured. The study did not account for elevated troponin levels, due to any type of kidney pathology or systemic conditions of hypotension [14]. Troponin concentrations in the blood have an inverse relationship to the estimated glomerular filtration rate (eGFR), if renal perfusion; or glomerular filtration decreases due to any inciting incidence, it can alter the troponin values collected [15,16]. A study conducted by Schmid et al. (2018) used high-sensitivity troponin T testing for patients with hereditary and acquired skeletal myopathies and found that 68.9% of patients tested above the 14 ng/L threshold for the assay [17]. One of many possible comorbidities that can affect the diagnostic workup for patients presenting acutely with a possible ACS event [17]. High-sensitivity troponin assays have allowed the detection of a predetermined level of troponins, but there is a lack of supporting data to rationalize what is a normal or abnormal level of troponin given specific comorbidities. The uncertainty of troponin level cut-offs can be appreciated in Figure 3, which demonstrates a wide range of troponin levels seen in patients who had the same diagnosis and outcome. This study suggests there is no difference in mortality or catheterization in different troponin assays but the sensitivity of the new generation of testing facilitates more false positive results when trying to diagnose an ACS event [17].

Limitations 

Uncontrollable factors could have influenced the results of the retrospective study. One was the sample size. Patient information was collected over several years, but only patients presenting with NSTEMI were included. The generalizability of findings to populations, and the population bias of the patients admitted to this hospital were from a very small region of south Miami. These features impact the external validity of findings to different populations, but this study is a data point when comparing cardiac catheterization and mortality in patients with a similar demographic bias to South Miami.

Further investigations into troponin levels, cardiac catheterization and mortality, should be conducted on a larger scale to determine potential sources of bias that could change future ACS management. Although no significant association was found between the type of troponin catheterization and mortality. A database of troponin levels measured in patients who were admitted for an ACS event with their associated ethnicity, and comorbidities could prove to be helpful in future ACS management framework.

## Conclusions

In this study, neither of the assays showed a more significant association with catheterization and mortality outcomes. Following guidelines, identifying core symptoms, normalizing the time between samples, and accurately measuring troponin values are vital to establish proper clinical conduct and disposition for future ACS events in any hospital. Further research is needed to evaluate how different comorbidities or variations in demographic values can impact troponin levels and ACS diagnoses.
